# Suture granuloma mimicking local recurrence of colon cancer after open right hemicolectomy: a case report

**DOI:** 10.1186/s40792-021-01251-2

**Published:** 2021-07-14

**Authors:** Shih-Feng Huang, Chia-Ling Chiang, Ming-Hung Lee

**Affiliations:** 1 Department of Surgery, Kaohsiung Veterans General Hospital, Kaohsiung, Taiwan, ROC; 2 Department of Radiology, Kaohsiung Veterans General Hospital, Kaohsiung, Taiwan, ROC; 3 School of Medicine, National Yang Ming Chiao Tung University, Taipei, Taiwan, ROC; 4 Division of Colorectal Surgery, Department of Surgery, Kaohsiung Veterans General Hospital, Kaohsiung, Taiwan, ROC

**Keywords:** Suture granulomas, Colon cancer, Postoperative surveillance, Recurrence, Right hemicolectomy

## Abstract

**Background:**

Foreign body granuloma is a rare surgery-related complication that can masquerade as cancer recurrence during postoperative surveillance. It may therefore deceive clinicians and lead to unnecessary interventions. The case presented herein demonstrates how a foreign body granuloma can be misleading in preoperative radiological studies and why this condition should not be ignored in differential diagnoses during surveillance of patients with previous history of abdominal surgery of any kind.

**Case presentation:**

We report a case of suture granuloma mistaken for recurrent colon cancer, including the clinical history, imaging data, and histopathological photographs. A 60-year-old man was followed up at our institution after open right hemicolectomy 2 years earlier for ascending colon carcinoma. Contrast-enhanced computed tomography and magnetic resonance imaging revealed an infiltrative heterogeneous soft tissue lesion at the right mesenteric root, adjacent to the ileocolic anastomosis. Local recurrence was therefore suspected. We performed exploratory laparotomy, excised the tumor, and sent it for histopathological examination, which confirmed suture granuloma.

**Conclusions:**

Foreign body granuloma is a rare surgery-related complication that should be considered during surveillance following colectomy. Its radiological features may mimic recurrent lesions, thus misleading clinicians and causing unnecessary interventions or further complications.

## Background

Postoperative surveillance following radical colectomy is vital to the successful treatment of colorectal cancers, and various imaging modalities are used for this purpose [1]. However, surgery-related foreign body granulomas can mimic tumors on imaging studies and can therefore mislead clinicians into applying unnecessary interventions [2–5].

In this report, we describe a rare case of a patient with a history of ascending colon carcinoma after right hemicolectomy, who developed a silk suture granuloma mistaken for local cancer recurrence.

## Case presentation

**Fig. 1:**
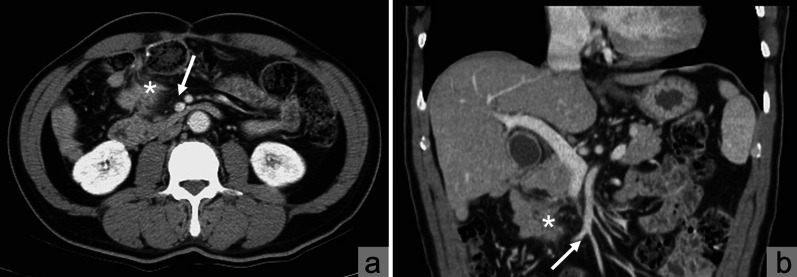
Preoperative contrast-enhanced CT study revealed **a** the relative position of the right mesenteric root (white arrow) and the lesion (white star sign) on axial view; and **b** the relative position of the right mesenteric root (white arrow) and the lesion (white star sign) on coronal view

**Fig. 2:**
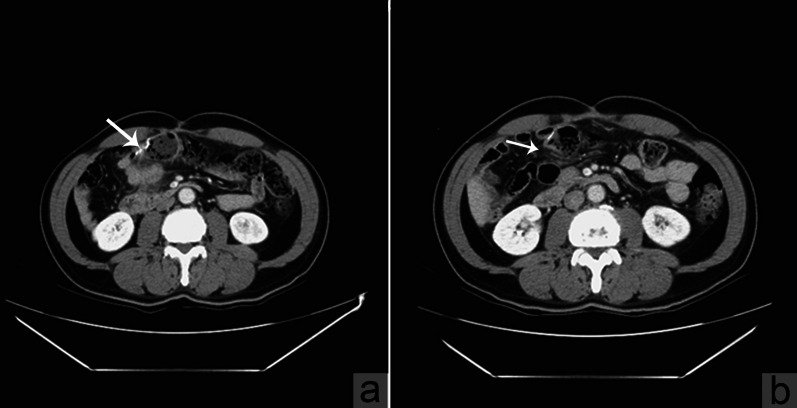
Contrast-enhanced abdominal CT study showed **a** a moderate contrast enhancement with apparent desmoplastic reaction near previous surgical sutures (white arrow); and **b** complete lesion clearance three months after surgery (white arrow)

**Fig. 3:**
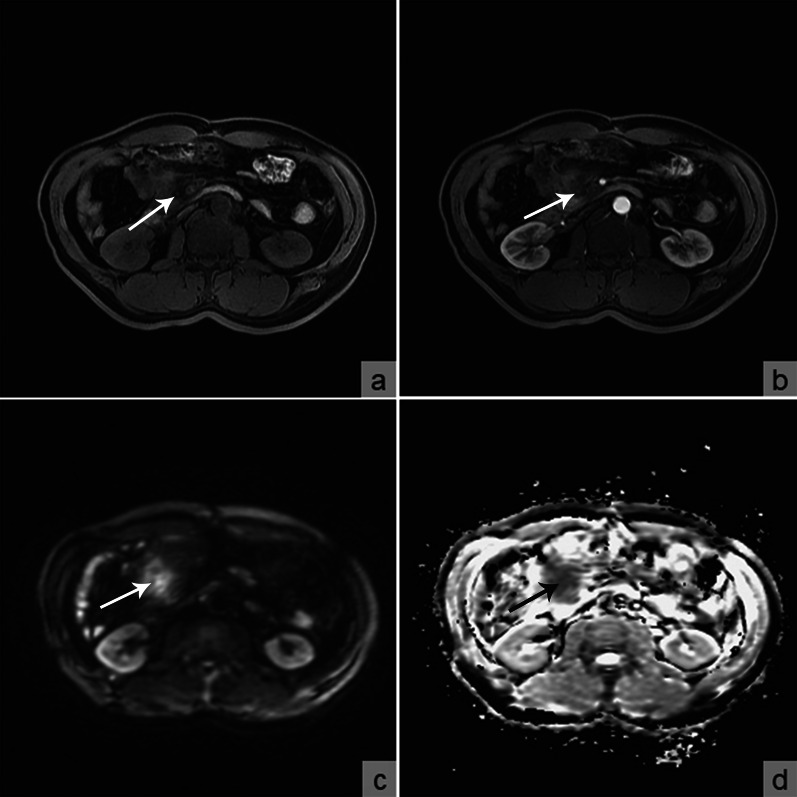
Abdominal MRI revealed a heterogeneous infiltrative soft tissue lesion near the previous ileum–colon anastomosis exhibiting **a** hypointensity on precontrast T2-weighted imaging (white arrow); **b** delayed and moderate enhancement on postcontrast T2-weighted imaging (white arrow); **c** hyperintensity on diffusion-weighted imaging (white arrow), and **d** water restriction on the ADC map (black arrow)

The patient was a 60-year-old man with a history of hypertension. He had been diagnosed with adenocarcinoma of the ascending colon with bowel obstruction (pT3N0M0, high risk stage II) and had undergone open right hemicolectomy approximately 2 years earlier. The surgery was done with ligation of right colic artery and ileocolic artery and vein. We encountered several small bleeders near the mesentery, and some of them were secured with silk suture ligations. We performed ileocolic anastomosis with hand-sewn technique with silk sutures and 3-0 Monocryl sutures. Oral tegafur and uracil had been administered as adjuvant chemotherapy, and no complications had been observed. After surgery, he had been regularly followed up at our institution every 3 months. However, recent abdominal computed tomography (CT) revealed a newly developed infiltrative heterogenous soft tissue lesion near the right mesenteric root (Fig. 1) and adjacent to the previous ileocolic anastomosis, causing regional tissue traction and small intestine adhesion (Fig. 2). Magnetic resonance imaging (MRI) performed 2 months later revealed lesion progression with water restriction on an apparent diffusion coefficient (ADC) map (Fig. 3). The lesion mimicked a pervasive tumor with desmoplastic reaction around it and the previous suture site. PET/CT was not covered by our national health insurance, and the patient did not have enough financial support to pay for a PET/CT examination. The patient denied fever, abdominal pain, nausea/vomiting, and other discomfort. However, tumor markers were elevated: the carcinoembryonic antigen (CEA) level was 2.50 ng/mL (3 months earlier 2.43 ng/mL), and the carbohydrate antigen 19-9 (CA19-9) level was 50.61 U/mL (3 months earlier 33.51 U/mL). Local recurrence was therefore suspected because of the radiological features associated with progression. We have discussed with the patient and offered an option of continuing close surveillance in addition to surgery. The patient favored the surgical option, and we consequently arranged tumor resection.

**Fig. 4:**
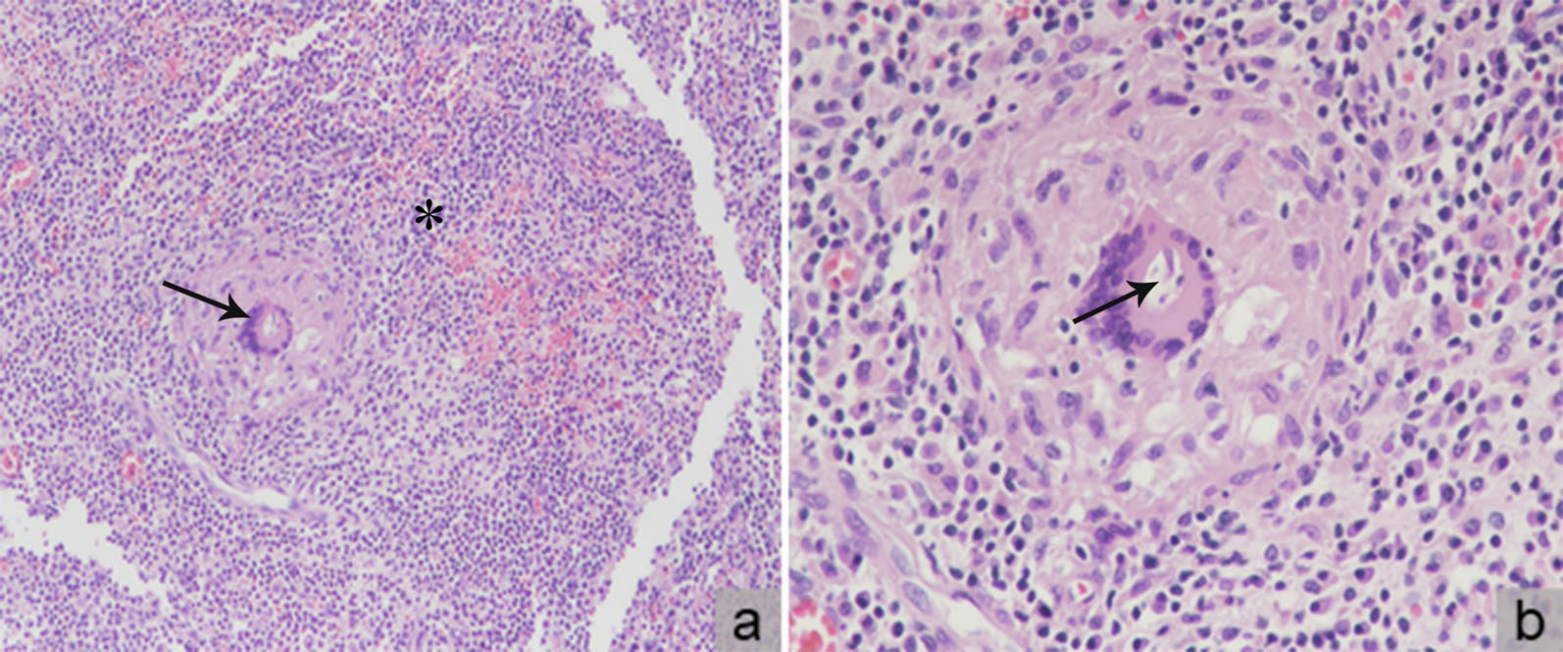
Histopathological examination revealed **a** chronic and acute inflammatory cell infiltration, fibrosis, necrosis, degenerative leukocyte aggregation (black star), foreign body-type giant cells, and granulomas (black arrow) under 100× magnification and **b** a multinucleated giant cell engulfing the suture material (black arrow) under 400× magnification

During surgery, we observed a 4 cm × 4 cm × 3 cm tumor arising from the mesentery near the previous ileocolic anastomosis, with involvement of the small intestinal wall approximately 50 cm from the ileocecal valve. We excised the tumor en bloc with a LigaSure® Vessel Sealing System (Medtronic, Dublin, Ireland) scalpel, including the part contacting the small intestinal wall, and then repaired the small intestinal wall defect. The specimen was sent for histopathological examination. Grossly, the tumor appeared grayish and included fragments of silk suture. Under a microscope, the specimen revealed an abscess and foreign body-type granulomatous inflammation. The tumor featured chronic and acute inflammatory cell infiltration, fibrosis, necrosis, aggregation of degenerative leukocytes, foreign body-type giant cells, and granulomas (Fig. 4). Neither mycobacteria nor fungi were identified with an acid fast or periodic acid–Schiff stain, and no evidence of malignancy was discovered. Seven days after surgery, the patient was discharged with no complications observed during the hospitalization.

## Discussion

A foreign body granuloma is a rare surgical complication, but easily neglected by surgeons. Foreign biomaterials such as sutures, clips, sponge and gauze fragments may be introduced into a patient’s body during surgery. These foreign materials may cause varying degrees of local immune response with giant cell reaction and cause clinical manifestations such as adhesions, tumor masses, or, as in this case, lesions mimicking tumor metastases or recurrence at a previous operative site [2–10]. Suture granulomas most commonly develop in response to nonabsorbable materials such as silk remaining in the patient’s body [10]. In the present case, a suture granuloma—caused by the silk thread used for vessel ligation or hand-sewn anastomosis—that mimicked colon carcinoma recurrence was identified.

Clinical manifestations of intra-abdominal foreign body granulomas are nonspecific and commonly appear as adhesions, abscesses, or malignancies, even after employment of various imaging modalities such as ultrasonography, CT, positron emission tomography (PET), or MRI [4, 5]. PET/CT was widely utilized for postoperative surveillance, however, several case reports showed that suture granulomas can cause false positivity on PET/CT. In cases of colorectal cancer, a false positive rate on PET/CT of 2–11% has been reported. This false-positive result may result from the inflammatory response aroused by the foreign body [6, 7]. Furthermore, elevation of tumor markers such as CEA and CA-199 does not exclude foreign body granulomas. There were plenty of case reports in the literature reporting foreign body granulomas with an elevation of tumor markers [7]. False-positive results of tumor makers during postoperative surveillance of colorectal cancer are not uncommon. A specificity of only 66.67% and 71.43% for CEA and CA-199, respectively, has been reported [11]. Given that imaging modalities and tumor markers could be unreliable, granulomas may be nearly indistinguishable from recurrent malignant lesions and thus affect the clinical judgments guiding subsequent treatment plans. Unnecessary interventions such as extended radical colectomy may be administered if a clinician does not consider a foreign body granuloma in the differential diagnoses and thus failing to recognize it. In our case, the suture granuloma was not confirmed until the final histopathological examination. The clinical history and radiological findings were deceptive and led to the wrong initial impression of locally recurrent cancer. In addition, intraoperative frozen section may help to guide judgment during surgery when the tumor in question lacks the gross characteristics typical of malignancy. If benign nature is reported by intraoperative frozen section, then an extensive radical surgery with or without lymph nodes dissection is not necessary. Nevertheless, a simple tumor resection is still reasonable to prevent further possibilities of development into severe local inflammation that may lead to severe adhesion or even bowel perforation [12] as long as there is no concern of high risks of perioperative complications such as large vessels or bowel wall injury. Further studies comparing the long-term outcomes of surgical tumor resection and watchful observation will be needed to make a valid conclusion. Suture granuloma is a differential diagnosis of considerable clinical relevance, especially in colorectal cancers because of the importance of intense surveillance following radical colectomy.

## Conclusions

Suture granuloma is a rare surgery-related complication that should be considered in patients with previous abdominal surgery of any kind. Diagnosing suture granuloma can be challenging because of its nonspecific clinical manifestations and radiological features, which make it difficult to distinguish from other conditions, such as adhesion, abscess, or malignancies. Successfully identifying a suture granuloma that appears as a malignant lesion may prevent further unnecessary intervention.

## Data Availability

Not applicable.
